# Transforming environmental health datasets from the comparative toxicogenomics database into chord diagrams to visualize molecular mechanisms

**DOI:** 10.3389/ftox.2024.1437884

**Published:** 2024-07-22

**Authors:** Brent Wyatt, Allan Peter Davis, Thomas C. Wiegers, Jolene Wiegers, Sakib Abrar, Daniela Sciaky, Fern Barkalow, Melissa Strong, Carolyn J. Mattingly

**Affiliations:** ^1^ Department of Biological Sciences, North Carolina State University, Raleigh, NC, United States; ^2^ Center for Human Health and the Environment, North Carolina State University, Raleigh, NC, United States

**Keywords:** data visualization, chord diagram, r, molecular mechanisms, environmental health, database, tetramers

## Abstract

In environmental health, the specific molecular mechanisms connecting a chemical exposure to an adverse endpoint are often unknown, reflecting knowledge gaps. At the public Comparative Toxicogenomics Database (CTD; https://ctdbase.org/), we integrate manually curated, literature-based interactions from CTD to compute four-unit blocks of information organized as a potential step-wise molecular mechanism, known as “CGPD-tetramers,” wherein a chemical interacts with a gene product to trigger a phenotype which can be linked to a disease. These computationally derived datasets can be used to fill the gaps and offer testable mechanistic information. Users can generate CGPD-tetramers for any combination of chemical, gene, phenotype, and/or disease of interest at CTD; however, such queries typically result in the generation of thousands of CGPD-tetramers. Here, we describe a novel approach to transform these large datasets into user-friendly chord diagrams using R. This visualization process is straightforward, simple to implement, and accessible to inexperienced users that have never used R before. Combining CGPD-tetramers into a single chord diagram helps identify potential key chemicals, genes, phenotypes, and diseases. This visualization allows users to more readily analyze computational datasets that can fill the exposure knowledge gaps in the environmental health continuum.

## 1 Introduction

Chemicals are important environmental factors that can affect human health, yet the molecular mechanisms connecting an exposure to an adverse outcome often remain unknown, reflecting knowledge and information gaps in the continuum (Thessen et al., 2020). The Comparative Toxicogenomics Database (CTD; https://ctdbase.org) is an innovative public resource that provides manually curated information from the scientific literature to harmonize and centralize cross-species data for chemical exposures and their interactions with genes, phenotypes (non-disease biological processes), and diseases (Davis et al., 2023a). Currently, CTD includes 3.5 million manually curated interactions relating 17,600 chemicals, 55,000 genes, 6,600 phenotypes, and 7,300 diseases. Additionally, CTD leverages data integration strategies to compute potential molecular mechanistic solutions that help fill knowledge gaps between exposure and adverse outcomes; presently, CTD generates over 88 million toxicogenomic relationships for users to explore (https://ctdbase.org/about/dataStatus.go). CTD datasets have been used to analyze diverse environmental health issues, including ozone-induced myocardial infarction (Davis et al., 2020), arsenic-linked male reproductive defects (Chai et al., 2021), heavy metal-associated depression (Nguyen and Kim, 2023), fatty liver disease resulting from perfluorooctane sulfonate (Kim et al., 2023), chemical-induced pulmonary fibrosis (Jeong et al., 2023), and hepatic toxicity from bisphenol exposure (Zhao et al., 2024).

**FIGURE 1:**
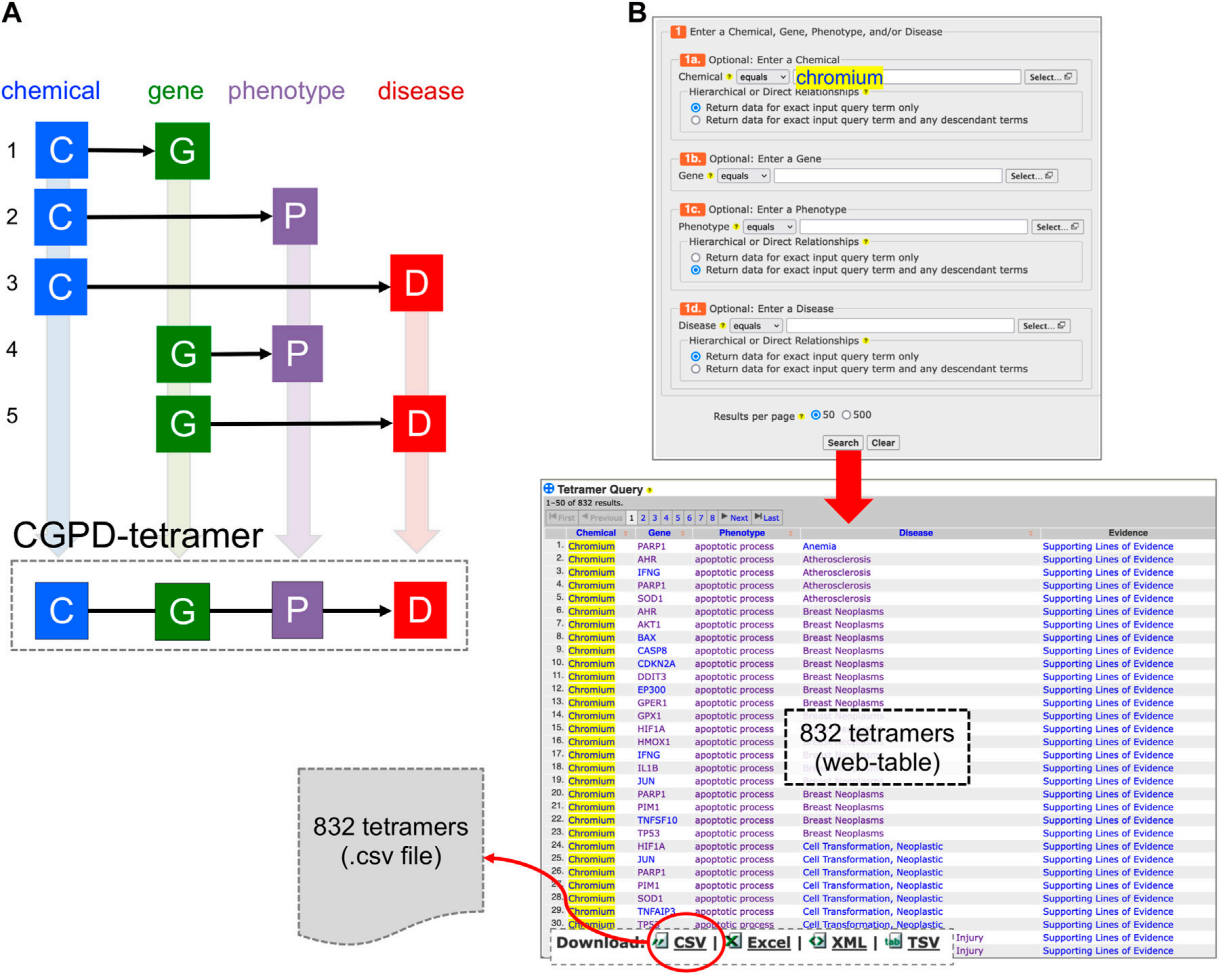
**(A)** A CGPD-tetramer is computationally generated by data integration only if five independently curated interactions (1–5) currently exist in CTD, interrelating a chemical (C), gene (G), phenotype (P), and disease (D). **(B)** A new analytical tool called “CTD Tetramers” (https://ctdbase.org/query.go?type=tetramer) enables users to generate their own tetramers by providing one or more chemical, gene, phenotype, or disease terms of interest. Here, “chromium” is entered as the chemical of interest, and CTD computationally generates 832 unique tetramers for this environmental metal with its associated gene, phenotype, and disease components. The query results are displayed as a sortable web-page table and can be downloaded as a file in different formats, including CSV.

Through data integration and novel analytical approaches, CTD has the capacity to provide insights into complex mechanistic relationships between chemical exposures and adverse outcomes by generating “CGPD-tetramers”: four-unit computational blocks of information that link an initiating Chemical with a Gene to modulate a Phenotype that can be inferred to a Disease outcome (Davis et al., 2020). To compute a tetramer, five independently, manually curated relationships in CTD are integrated, including a chemical-gene interaction, a chemical-phenotype interaction, a chemical-disease interaction, a gene-disease interaction, and a gene-phenotype annotation (Figure 1A). A new analytical tool called “CTD Tetramers” (Davis et al., 2023b) allows users to generate CGPD-tetramers for any chemical, gene, phenotype, or disease of interest (Figure 1B). However, most queries typically return hundreds or thousands of tetramers, displayed as a web-based table, wherein the content can be sorted or downloaded.

**FIGURE 2:**
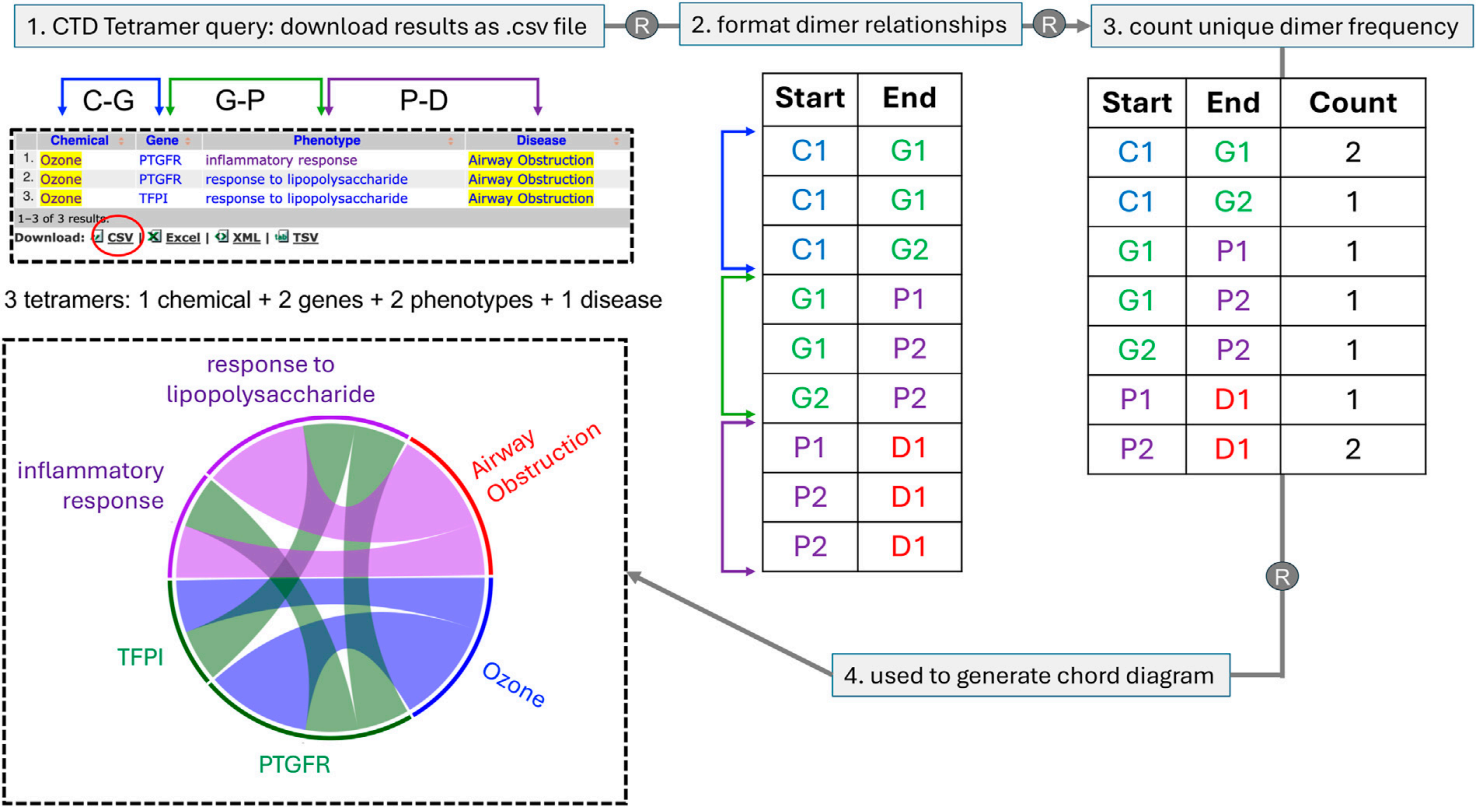
A schematic of how multiple CGPD-tetramers are converted into a single chord diagram. Here, for simplicity, only three tetramers are used, involving one chemical, two genes, two phenotypes, and one disease that are displayed as a web-table, and can be downloaded as a file in CSV format. Using R, individual dimer relationships between unique chemicals (C), genes (G), phenotypes (P), and diseases (D) in the tetramer set are first formatted; then, the number of unique dimer frequencies are counted and this value is used to scale the corresponding length of the node and width of the connecting arcs for each data type. Chord diagrams offer a more pleasing visual representation of the same data seen in a table, but enables key elements to be more easily recognized due to the scaled length and width of the nodes and arcs, respectively. Diagrams are read in a clockwise direction, in the same order that a CGPD-tetramer is read: here, the initiating chemical (Ozone) interacts with two genes (PTGFR and TFPI), which in turn modulate two phenotypes (inflammatory response and response to lipopolysaccharide), ending in the disease airway obstruction. The scaled dimensions of the nodes and arcs reflect the frequency of the terms in the total tetramer set, allowing potential key mechanisms to be more easily identified (here, ozone, PTGFR, and response to lipopolysaccharides are overrepresented in this simplistic illustration). The four data types are color-coded to facilitate demarcation in the diagram, using a blue palette (for chemicals), green palette (for genes), purple palette (for phenotypes), and red palette (for diseases).

To extend the analysis of these large CTD Tetramer query results, we describe a novel method to transform CGPD-tetramers into a chord diagram that allows patterns and relationships to be more easily recognized. The diagram links information in a circular layout by listing the data types as nodes around the circumference of the circle and using arcs to depict the connecting relationships among the nodes. Each time a relationship is plotted, the corresponding node gets larger, and as nodes and arcs grow in length and width, it becomes visually apparent which components occur most frequently (Figure 2). This approach allows all the CGPD-tetramers from a table to be combined and visualized in a single graph, facilitating the identification of potentially key mechanistic elements connecting a chemical exposure to a disease endpoint.

The chord diagrams are compact, elegant representations of complex tetramer datasets. To generate these diagrams, we make use of R, a widely used and publicly available programming environment that can be run on all major operating systems, including Windows, MacOS, and Linux. The R-based script and instructions that we provide are easy to learn, so that even novice users can quickly begin creating chord diagrams illuminating CTD tetramer results.

## 2 Methods

### 2.1 CTD data version and analysis

Analysis was performed using CTD public data available (revision 17,266; March 2024). CTD is updated with new content on a monthly basis (https://ctdbase.org/about/dataStatus.go); consequently, query results described in this text may vary over time.

### 2.2 R environment

The freely available version 4.4.0 of R (https://www.r-project.org) was used (R Core Team, 2018). The R package data.table version 1.15.2 (https://CRAN.R-project.org/package=data.table) was used in preparing the data and the R package circlize version 0.4.16 (https://CRAN.R-project.org/package=circlize) was used to generate the chord diagrams (Gu et al., 2014). To format CGPD-tetramers and plot a basic chord diagram, download the *CTD-vizscript.R*, from GitHub (https://github.com/CTD-NCSU/Chord_Diagrams) or use the version included with a dataset of test tetramers (“test.csv”) for practice (Supplemental File S1). For CTD users unfamiliar with R, it is not necessary to download local versions of the software to generate the chord diagrams. Instead, a free, web-based version of R and RStudio environment from Posit Cloud (https://posit.cloud) may be used to generate the diagrams; users will need to first create a free account at Posit Cloud, and the steps required using the latter method are provided below.

### 2.3 Basic steps for CTD tetramer visualization using web-based Posit Cloud

Step 1: Download CTD Tetramers query results in CSV format (and rename the file to something more intuitive); start with a dataset of no more than 1,500 tetramers (discussed below); alternatively, use the test sample CSV file provided as “test.csv” (Supplemental File S1), which contains 309 CGPD-tetramers composed of two chemicals (copper and iron), 61 genes, 87 phenotypes, and 1 disease (liver neoplasms).

Step 2: Download *CTD-vizscript.R* locally from GitHub (https://github.com/CTD-NCSU/Chord_Diagrams) or use the provided file (Supplemental File S1)

Step 3: Create a free account at Posit Cloud (https://posit.cloud) and log in to the “Your Workspace” web page.

Step 4: On the right-hand side of the workspace web page, click on the “New Project” button and select “New RStudio Project” from the drop-down menu. The browser will reload showing three panes: “Console” in the upper left, “Environment” in the upper right, and “Files” in the bottom right; browser screenshots for the subsequent steps are provided (Supplemental File S2). In the bottom right pane (“Files”), click on the “Upload” button to browse for and select the downloaded copy of *CTD-vizscript.R* file (panel A, Supplemental File S2); then upload the CSV formatted file of the CTD Tetramer query results that are to be visualized, or use the provided sample file *“*test.csv.*”* Both files will now be listed in the “Files” pane.

Step 5: Click on the *CTD-vizscript.R* file and the script will appear in a new upper left-hand pane (and “Console” will displace to the bottom left). An alert will pop up and ask to install circlize and data.table. Choose install (panel C, Supplemental File S2). In the “Console” window, you’ll see the program run until it is finished.

Step 6: Load data.table and circlize using the library() command to make them available for future steps. To do this, go to code lines 2–3 and execute the two lines of the script by using the cursor to physically highlight the two lines for library(data.table) and library(circlize) in the window pane, and then click “Run” button (panel D, Supplemental File S2).

Step 7: Under “#Import data,” at code line 6 assign the name of the CTD Tetramer query result file to the variable “tetramers” (panel E, Supplemental File S2). To do this, type in the exact name of the file, which is case-sensitive, surrounded by quotation marks, and suffixed with “.csv,” all enclosed with the parentheses. For example, if using the provided test file (test.csv), then set the line to read: tetramers < - read.csv(“test.csv”). The case-sensitive file name must be spelled exactly, contain the CSV suffix, be flanked by quotation marks, and enclosed in the parentheses.

Step 8: Using the cursor, highlight the line from step 7 (where the tetramer file name is entered on code line 6) along with the rest of the script to code line 60 (panel F, Supplemental File S2). Click the “Run” button.

Step 9: If everything runs successfully, the tool will generate a colored chord diagram in the lower right pane under “Plots” (panel I, Supplemental File S2). This process can take a few minutes, depending upon the total number of CGPD-tetramers that are being graphed. See below for common errors that might arise. To adjust the font size of the labels, go to code line 59 and find “cex = ” near the end of the script under “## Add labels to diagram” (panel H, Supplemental File S2). “cex” stands for “character expansion” and is the parameter that changes the font size of the node labels. It has a default value of 1; to make the font size smaller, the parameter can be changed to a value between 0 and 1; for example, cex = 0.5 will result in text that is 50% smaller than the default value. Changing the parameter to a value greater than 1 will result in larger text; for example, cex = 1.5 will result in text that is 50% larger than the default value. The greater the number of tetramers being plotted, the smaller the value of cex should be in order for the labels to be clearly separated and not overlapping. Additionally, or alternatively, for very large sets of tetramers where the node labels are difficult to resolve, a user might instead prefer to display only the node accession identifiers, as discussed below. After changing the value of cex, go to code line 45 and highlight the text beginning with circos.clear along with the remaining script, and click “Run”. This is necessary to clear out the old data and rerun the program using the newly entered values.

Step 10: The chord diagram can be saved as either a PDF or an image by clicking the “Export” button under the “Plots” tab of the lower right-hand pane (panel I, Supplemental File S2).

### 2.4 Fixing common errors that might be encountered

Common error 1: An error that states Error in setDT(cgpd): could not find function “setDT” indicates the data.table package is not loaded. Highlight the line that says library(data.table) near the top of the script (code line 2) and click “Run.” This will make the function “setDT” available.

Common error 2: An error that states any of the following are not found, “circos.clear,” “circos.par,” “chordDiagram,” or “circos.track, indicates the circlize package is not loaded. Highlight code line 3 that says library(circlize) and click “Run.” This will make those functions available.

Common error 3: An error that states “Maybe your “gap.degree” is too large so that there is no space to allocate sectors” indicates that there is a very large number of tetramers. First, highlight the code line 45 that says circos.clear() and click “Run.” Next, go to code line 47 that says circos.par(gap.degree = 1) and change gap.degree to a number less than 1 (panel G, Supplemental File S2). This is a setting for the gaps between neighboring nodes. Larger values for gap.degree will create larger spacing between nodes and smaller values for gap.degree will create smaller spacing between nodes. Once gap.degree is changed to a smaller value, highlight the line along with the rest of the script and click “Run.” If the error occurs again, repeat the process of running circos.clear, changing gap.degree to an even smaller number and rerunning the script.

### 2.5 Customizing chord diagrams

To enhance the readability of large chord diagrams, it is sometimes preferable to shorten the node term label to an accession identifier (ID), as this further compacts the diagram by reducing the text. A label can be switched from “node term” to “node ID” easily by adding “.ID” as a suffix the end of the Chemical, Gene, Phenotype, or Disease variables (code lines 10–13) in the section under “# Prep data” (panel J, Supplemental File S2). Highlight the script beginning with this line along with the remaining script and click “Run”. Additional customization options can be found in the circlize documentation at https://jokergoo.github.io/circlize_book/book/index.html.

The default selection of color palettes for CTD tetramer chord diagrams are blue (chemical), green (gene), purple (phenotype), and red (disease). If necessary, users can change these colors using code lines 37–40 (panel K, Supplemental File S2). Each line of script for each of the four variables contains two colors. The script will create a palette from the first color listed to the second color. To change the color, simply edit the name in the code line, making sure the new color name is in quotation marks and the two colors are separated by a comma. There are many R color cheat sheets that can be found with an internet search to aid in choosing colors, including those that are specifically designed for users with color-vision deficiencies (Dahl et al., 2022).

A summary of the entire CTD-vizscript code is provided with information boxes showing the exact code lines that users can edit to customize the chord diagram output (panel L, Supplemental File S2).

## 3 Results

### 3.1 Visualizing a single dataset of tetramers

**FIGURE 3:**
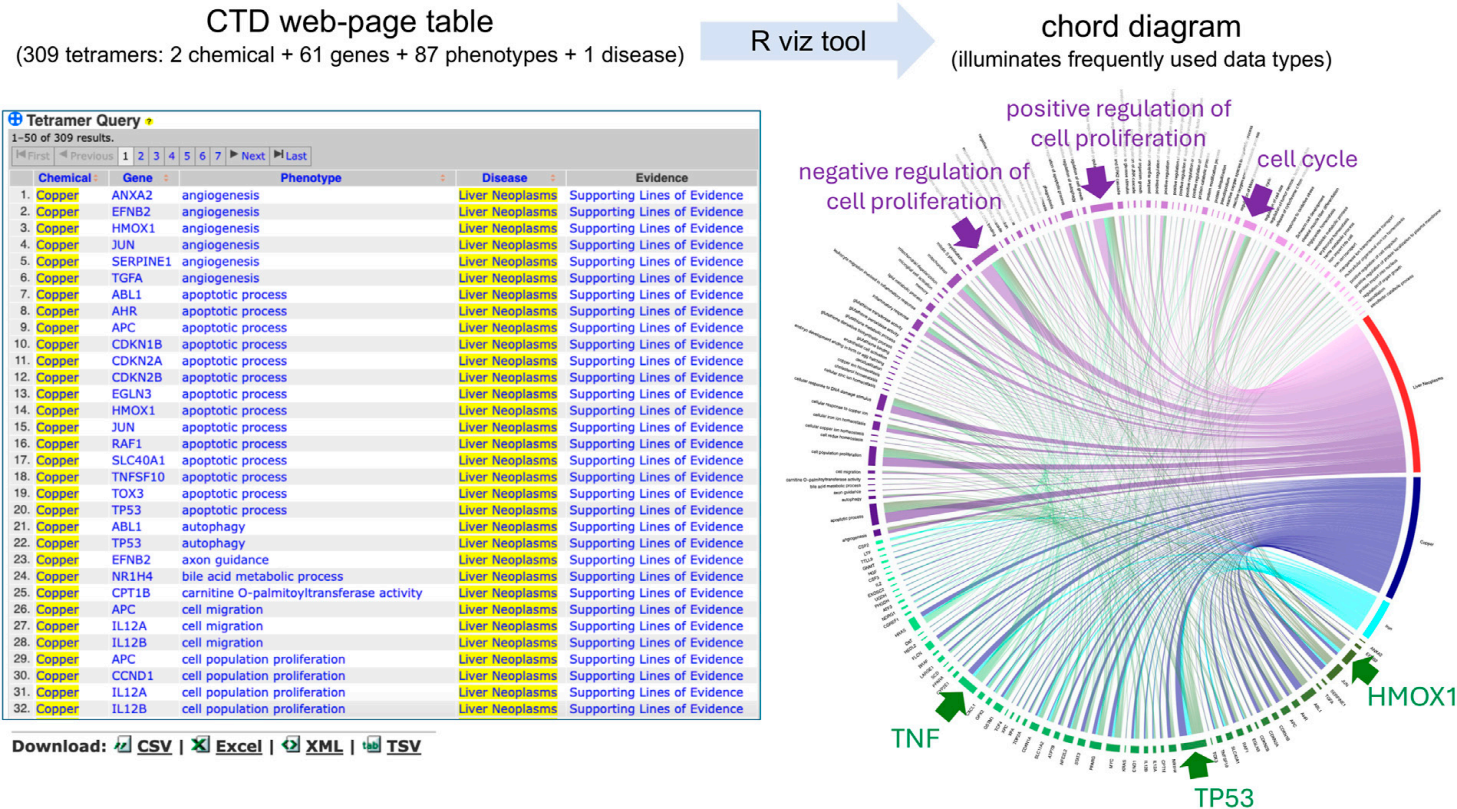
CTD Tetramer query results are returned as a web-page table, and the default display shows only the first 50 tetramers. Here, 309 tetramers relate two metals (copper and iron) with 61 genes and 87 phenotypes to the disease outcome of liver neoplasms. To survey all 309 tetramers on the web page, the user can scroll and click open additional web-pages or sort the pages by clicking on column headers. Our visualization method enhances this analysis by transforming and depicting all 309 CGPD-tetramers in a single chord diagram, allowing the user to more readily identify potentially key components of the dataset, due to the scaled dimensions of nodes/arcs in the graph, such as the indicated genes (TNF, TP53, and HMOX1) and phenotypes (cell proliferation and cell cycle). Nodes and their projecting arcs represent the directionality, are read clockwise, and are color-coded, initiating with chemicals (blue palette) to genes (green palette) to phenotypes (purple palette) and ending with diseases (red palette).

To demonstrate how to transform a large dataset of CGPD-tetramers into a single chord diagram, we queried the CTD Tetramers tool (https://ctdbase.org/query.go?type=tetramer) using “metals” as the chemical input, selecting the hierarchy filter to include any descendant terms, and “liver neoplasms” as the disease outcome. This yields 309 CGPD-tetramers, composed of two chemicals (copper and iron), 61 genes, and 87 phenotypes connected to liver neoplasms (Figure 3). The web-based table result displays only the first 50 of the 309 CGPD-tetramers (but it can be set to display 500). The tabular output is downloaded from the webpage as a CSV file, and then following the step-by-step directions (see Methods), the CTD Tetramer results are processed to generate a chord diagram that depicts all 309 CGPD-tetramers in a single image (Figure 3). Each chemical, gene, phenotype, and disease is represented as a colored node on the circumference of the circle, with chemicals shown in a blue palette, genes in a green palette, phenotypes in a purple palette, and diseases in a red palette. The arcs in the center of the circle connect the four nodes of each CGPD-tetramer, and are read in a clockwise manner: chemical (blue arc) to gene, gene (green arc) to phenotype, and phenotype (purple arc) to disease. The length of the node reflects the frequency that the specific data term occurs in the CGPD-tetramers and the width of the arc is proportional to the frequency that a dimer interaction occurs (Figure 2). Larger nodes/arcs readily highlight the more commonly used data types, and visually indicate potentially important molecular players, such as the genes HMOX1, TP53, and TNF, and the phenotypes cell proliferation and cell cycle for metal-associated liver neoplasms (Figure 3).

### 3.2 Combining and visualizing multiple datasets of tetramers

Environmental health is a complex, multifactorial system wherein outcomes can be the result of chemical mixtures and of multiple, independent chemical exposures from different sources (Davis et al., 2023b). Thus, CTD users often query for and analyze different sets of CGPD-tetramers for different chemicals and then combine the data to look for overlaps between shared molecular mechanisms and diseases. To aid in such analysis, multiple tetramer query results can be combined into a single chord diagram. To merge query results, it is most straightforward to download the individual CSV files for each independent query, combine the files into a single CSV document (using Excel copy-and-paste), and then upload that single file as the data source for generating a chord diagram as described above.

**FIGURE 4:**
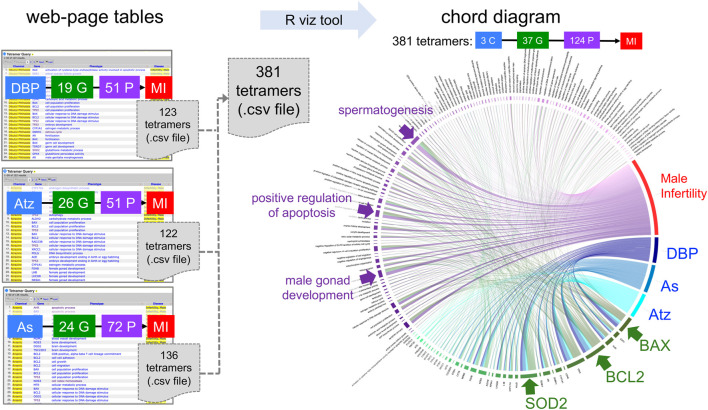
Multiple sets of CTD tetramer query results can be combined into a single chord diagram. Here, dibutyl phthalate (DBP), atrazine (Atz), and arsenic (As) are independently queried for their association with male infertility (MI), resulting in three separate datasets of 123, 122, and 136 CGPD-tetramers for each chemical-disease query, respectively. The three outputs can be manually combined by the user into a single CSV file of 381 total tetramers. The visualization tool transforms the merged datasets into a single chord diagram, and the scaled dimensions of nodes/arcs help identify potentially important genetic (BAX, BCL2, and SOD2, green arrows) and phenotypic (male gonad development, apoptosis, and spermatogenesis, purple arrows) molecular mechanisms.

For example, numerous environmental factors have been associated with male infertility, including phthalates, pesticides, and arsenic (Rodprasert et al., 2023). Three independent queries for CTD tetramers that relate the chemicals dibutyl phthalate, atrazine, and arsenic to male infertility can be combined into a single chord diagram to help identify potentially key genetic and phenotypic molecular mechanisms associated with these environmental stressors (Figure 4). In total, these three independent queries yield 381 CGPD-tetramers composed of 37 genes and 124 phenotypes connecting the three chemicals to male infertility. Visualization of this complex environmental health dataset illuminates candidate genes (BAX, BCL2, and SOD2) and phenotypes (male gonad development, apoptosis, and spermatogenesis) connecting the chemicals to male infertility, and thus may represent critical intermediate molecular mechanisms (Figure 4).

### 3.3 Limitations

An impediment for visualizing CGPD-tetramers as a chord diagram is spacing constraints that limit the comprehensibility of these diagrams. We have found that about 1,500 tetramers tend to represent a practical upper limit for figure clarity. For larger datasets, there are a few options. As described above, customizing the node displays either using the “cex” parameter (for font size) or changing the label from “term” to “node ID” will significantly reduce the text (panel J, Supplemental File S2), resulting in a more compact diagram, but still enabling nodes to be readily identified. As well, the starting number of CGPD-tetramers to be graphed can be reduced preemptively by adding additional search parameters in the query to narrow the dataset to focus on a more specific question. For example, a query for “liver neoplasms” results in over 16,700 CGPD-tetramers, and represents a very broad open-ended question with respect to environmental health. However, when “metals” is included as the chemical parameter in the search, the number reduces to 309 (Figure 3), and focuses on a more specific, addressable environmental health question. Alternatively, including phenotypes (e.g., “nervous system process,” “inflammatory response”, or “metabolic process,” with the filter option actively changed to include any descendant term) can also help focus questions and provide a more manageable number of CGPD-tetramers for analysis. Finally, CTD is also exploring ways to rank tetramers with a metric score as a means to prioritize the query results with the CGPD-tetramers that have the most supporting lines of evidence from the literature. Currently, the default display is alphabetical on the web-page results. Ranking would allow users to select tetramer subsets (instead of using the entire output) to include in their chord diagram.

Another limitation is the use of colors to represent information, as this may be an issue for those with color-vision deficiencies. Users can customize the plot colors to provide accessibility for specific color-vision deficiencies (Dahl et al., 2022) or for aesthetic purposes (panel K, Supplemental File S2).

Finally, the obvious complicating factor to this approach is the manual effort required to download and transfer data files from CTD to an R environment in order to generate a chord diagram. Consequently, we plan to integrate similar functionality directly into CTD Tetramer query results pages in a future release.

## 4 Discussion

Advances are being made to provide clarity to complex environmental health data, including development and adoption of standardized vocabularies (Mattingly et al., 2016; Holmgren et al., 2023) and development of visual frameworks that display, organize, and integrate data from diverse sources. For example, the Aggregate Exposure Pathway (AEP) collates and structures chemical and biological knowledge at the population level to connect an environmental stressor with its source, medium, external/internal concentrations, and target site exposure (Teeguarden et al., 2016), while the Adverse Outcome Pathway (AOP) assembles information at the cellular level to link an initiating molecular event, a series of key processes, and an adverse outcome (Ankley and Edwards, 2018). Together, the AEP and AOP frameworks complement each other and connect population data to molecular data, respectively, helping to bridge the environmental health continuum (Hines, et al., 2018).

To harmonize environmental health data, CTD embraces community-accepted FAIR controlled vocabularies and ontologies to curate the scientific literature for both population exposure science (Grondin et al., 2016) and molecular/cellular laboratory results (Davis et al., 2011). This imperative step enables disparate data to be integrated to generate inferred relationships that provide potential mechanistic solutions for recognized knowledge gaps (Davis et al., 2008). CGPD-tetramers are a novel derivative of this integration strategy, where the combined data types are structured as a step-wise mechanistic pathway: a chemical first interacts with a gene to then modulate a phenotype that may contribute to a final disease outcome (Davis et al., 2020). Users can easily generate CGPD-tetramers for any environmental chemical, gene, phenotype, or disease of interest at CTD (Davis et al., 2023b); however, these queries typically bring back hundreds of results, in a web-based table.

To help survey and distill these large environmental health datasets, we present a simple visualization method that transforms CGPD-tetramers into a chord diagram, allowing all of the data to be condensed and viewed in a single, elegant figure. CGPD-tetramers are formatted and transformed using R. While our technique can be easily adopted by researchers already familiar with R, we provide step-by-step instructions for novice users, leveraging a free web-browser system that enables inexperienced users to generate publication-worthy chord diagrams for their own presentations. By their nature, chord diagrams show the interconnections between different data types and facilitate the visual identification of the most frequently used data components, allowing users to discern potentially important intermediate molecular mechanisms between an environmental exposure and a disease outcome. Graphing these complex, toxicology-related mechanistic pathways can help build, inform, or refine current AOPs (Davis et al., 2023b) and be used to provide visual mechanistic links to AEPs. Together, both the table-based version of CGPD-tetramers and the derived chord diagrams complement each other, with the former providing data in an easily sortable format and the latter providing a graphical view that helps to visually detect key data types.

## Data Availability

The datasets presented in this study can be found in online repositories. The names of the repository/repositories and accession number(s) can be found in the article/Supplementary Material.
