# Supplementary material for: Transforming environmental health datasets from the comparative toxicogenomics database into chord diagrams to visualize molecular mechanisms
Source: Front Toxicol. 2024 Jul 22;6:1437884. doi: 10.3389/ftox.2024.1437884 (PMC11298510; doi:10.3389/ftox.2024.1437884)
Supplement: Supplementary file 2 [file DataSheet1.pdf]

SUPPLEMENTAL FILE S2

Supplemental File S2: Screenshots of posit Cloud web application.

Panel A: Uploading CTD-vizscript and tetramer CSV file.

Panel B: Click on CTD-vizscript to load script.

Panel C: Click on “Install” to load necessary packages.

Panel D: Use cursor to highlight just the first two lines of library packages, and click “Run”.

Panel E: Add correct name of the CSV file containing the tetramer data set; make sure the file name is flanked by parentheses.

Panel F: Use cursor to highlight all of the remaining script and click “Run”.

Panel G: A “gap degree” error means you need to reduce this value.

Panel H: Font size can be adjusted by editing “cex”.

Panel I: The generated chord diagram will appear under the “Plots” tab can be exported as a file.

Panel J: To change the node label text from “terms” to “accession identifiers”, simply append the suffix “.ID” to any of the Chemical, Gene, Phenotype, or Disease variables.

Panel K: To change the plot colors simply enter new R color terms (available from the web).

Panel L: The complete CTD-vizscript code highlighting the lines where users can make modifications.

A.

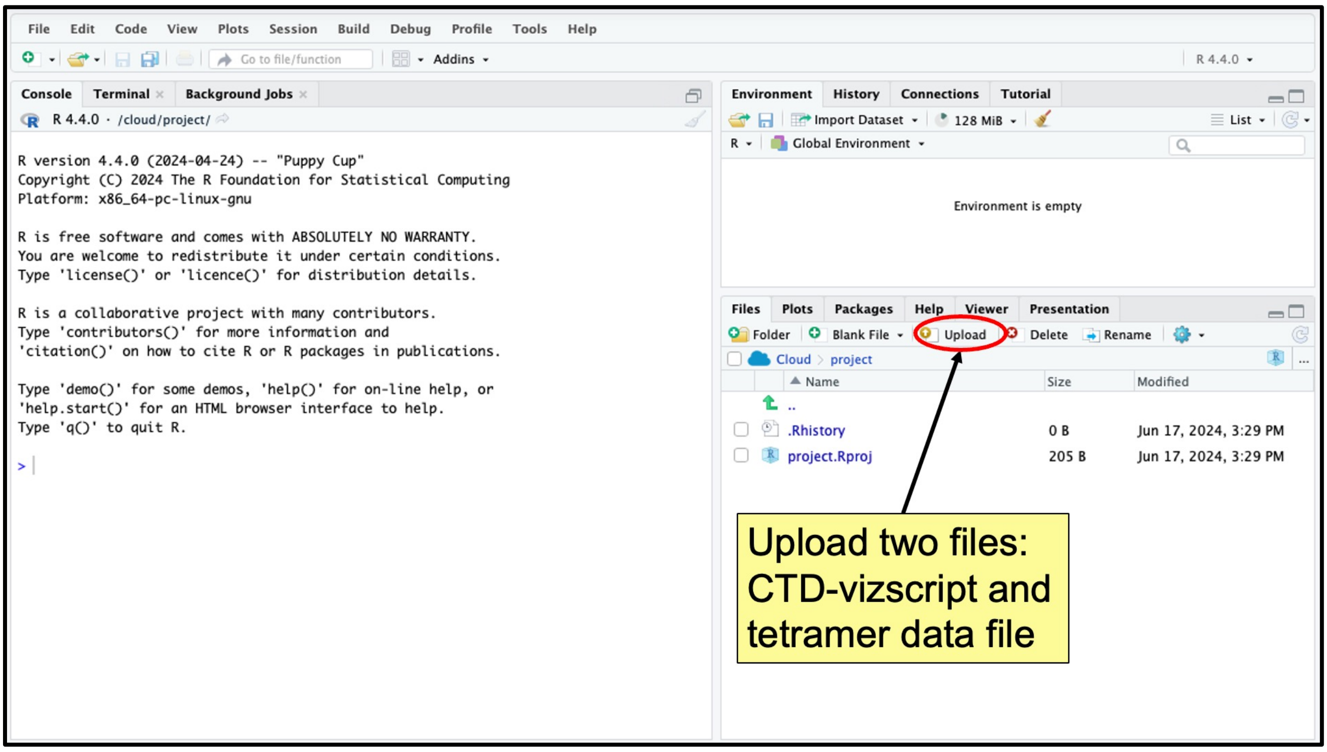
Upload two files: CTD-vizscript and tetramer data file
Upload
Environment is empty
.Rhistory
project.Rproj
R version 4.4.0 (2024-04-24) -- "Puppy Cup"

B.

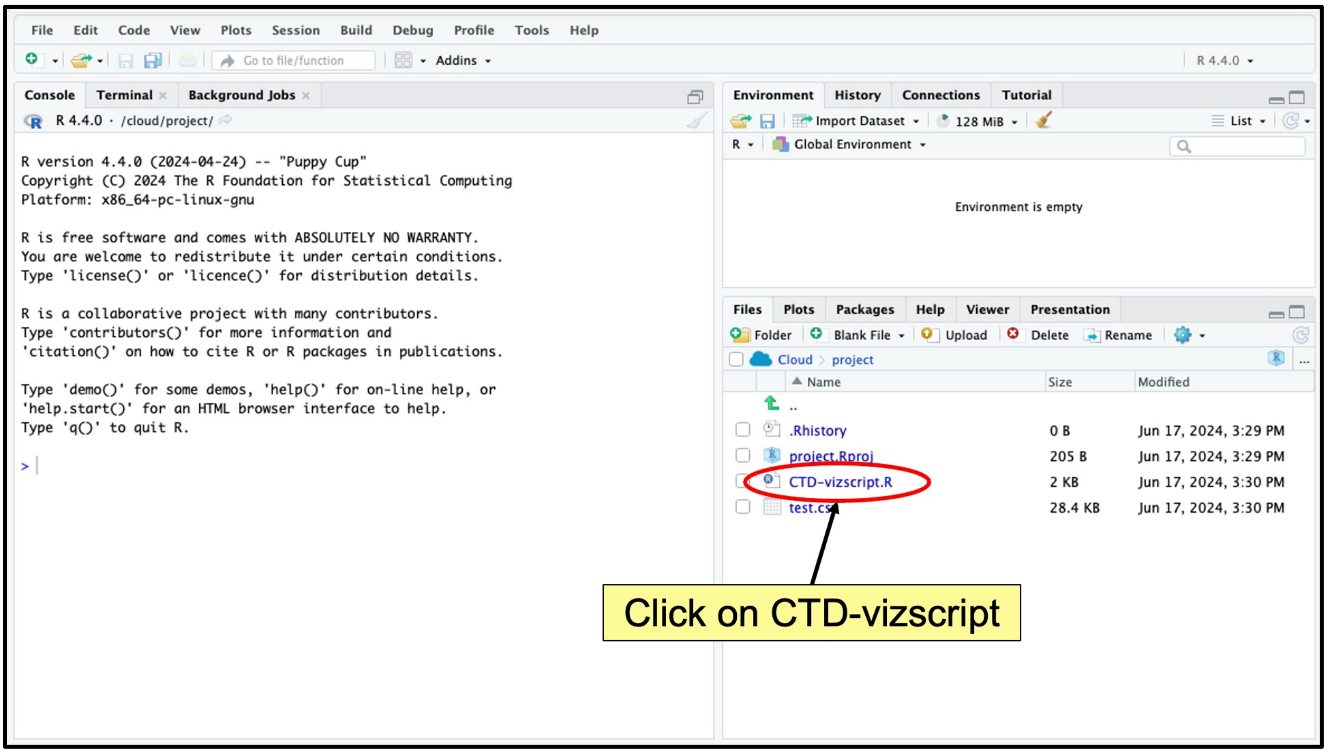
Click on CTD-vizscript
CTD-vizscript.R
test.csv
.Rhistory
project.Rproj

C.

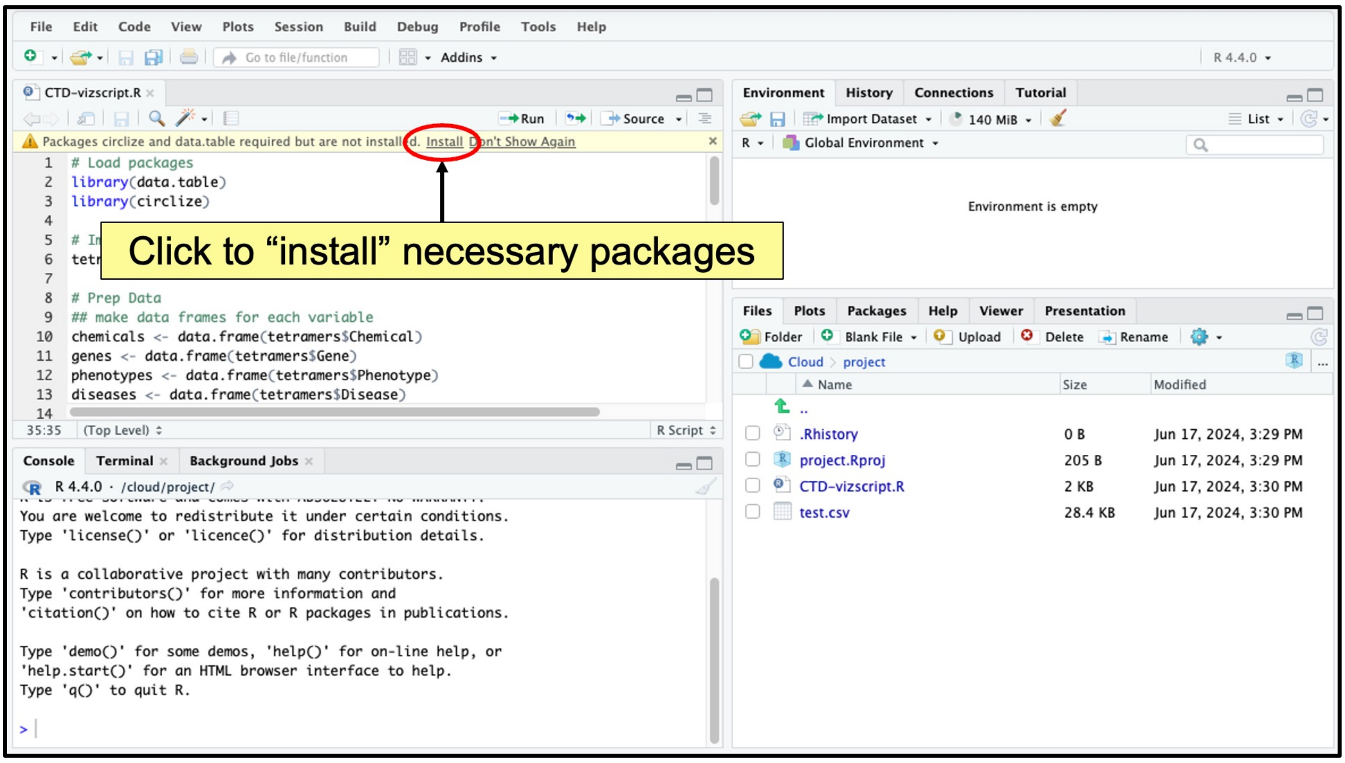
Click to “install” necessary packages
Packages circlize and data.table required but are not installed. Install Don't Show Again
# Load packages
library(data.table)
library(circlize)
# Prep Data
make data frames for each variable
chemicals <- data.frame(tetramers$Chemical)
genes <- data.frame(tetramers$Gene)
phenotypes <- data.frame(tetramers$Phenotype)
diseases <- data.frame(tetramers$Disease)
CTD-vizscript.R
test.csv

D.

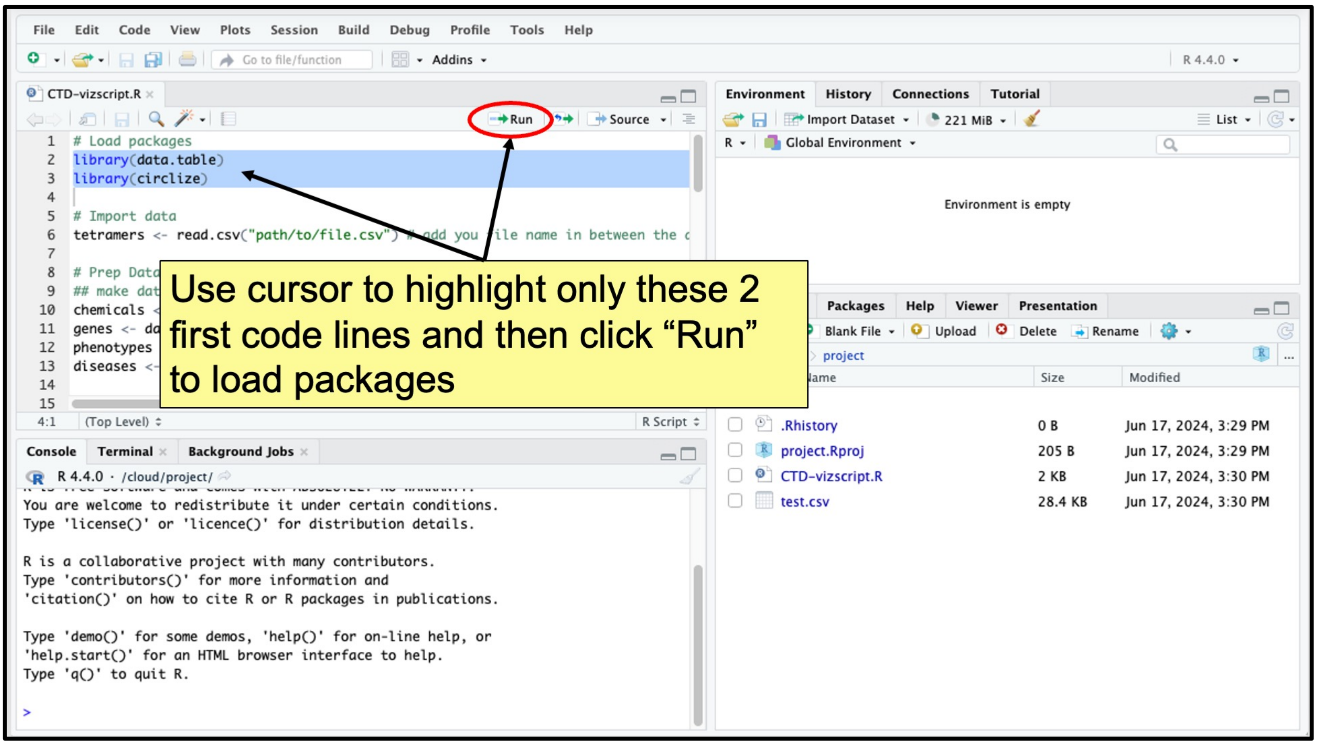
Run
# Load packages
library(data.table)
library(circlize)
# Import data
tetramers <- read.csv("path/to/file.csv") # add you file name in between the
Use cursor to highlight only these 2 first code lines and then click "Run" to load packages

E.

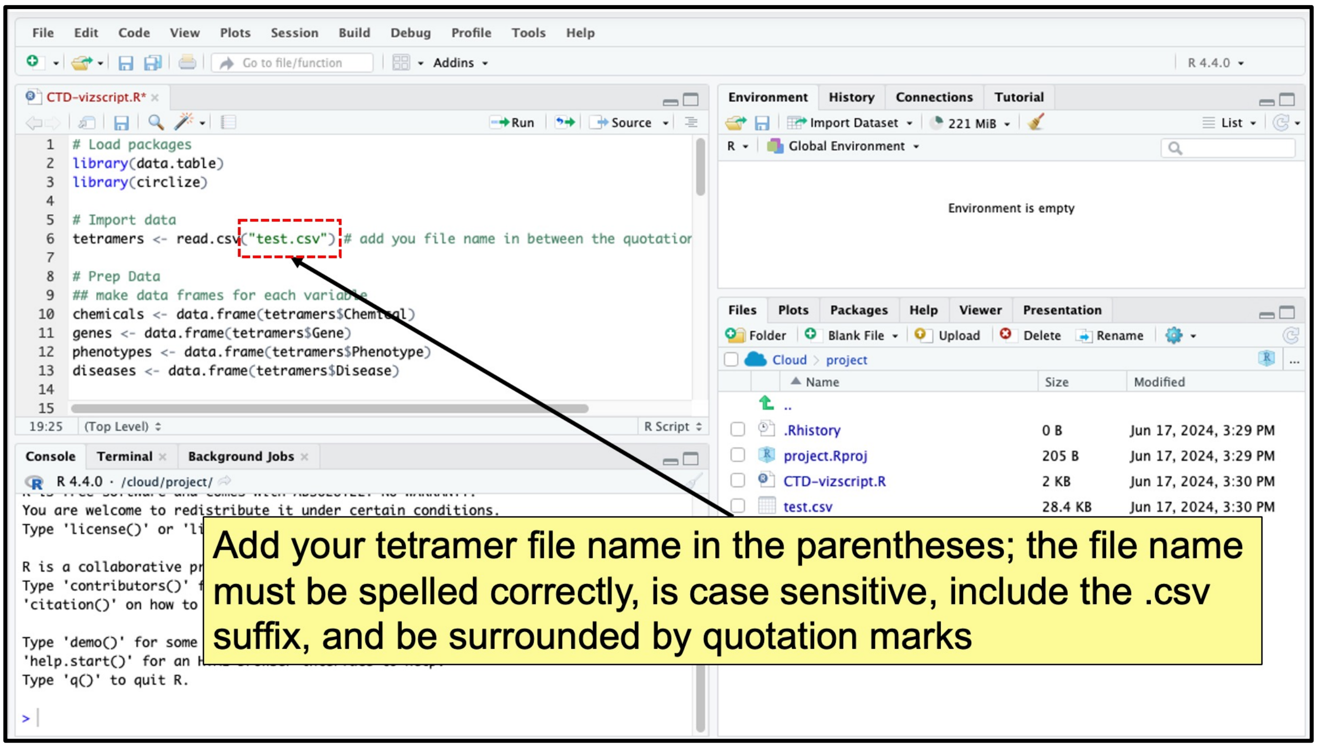
# Load packages
library(data.table)
library(circlize)
# Import data
tetramers <- read.csv("test.csv") # add you file name in between the quotatior
# Prep Data
make data frames for each variable
chemicals <- data.frame(tetramers$Chemical)
genes <- data.frame(tetramers$Gene)
phenotypes <- data.frame(tetramers$Phenotype)
diseases <- data.frame(tetramers$Disease)
Add your tetramer file name in the parentheses; the file name must be spelled correctly, is case sensitive, include the .csv suffix, and be surrounded by quotation marks

F.

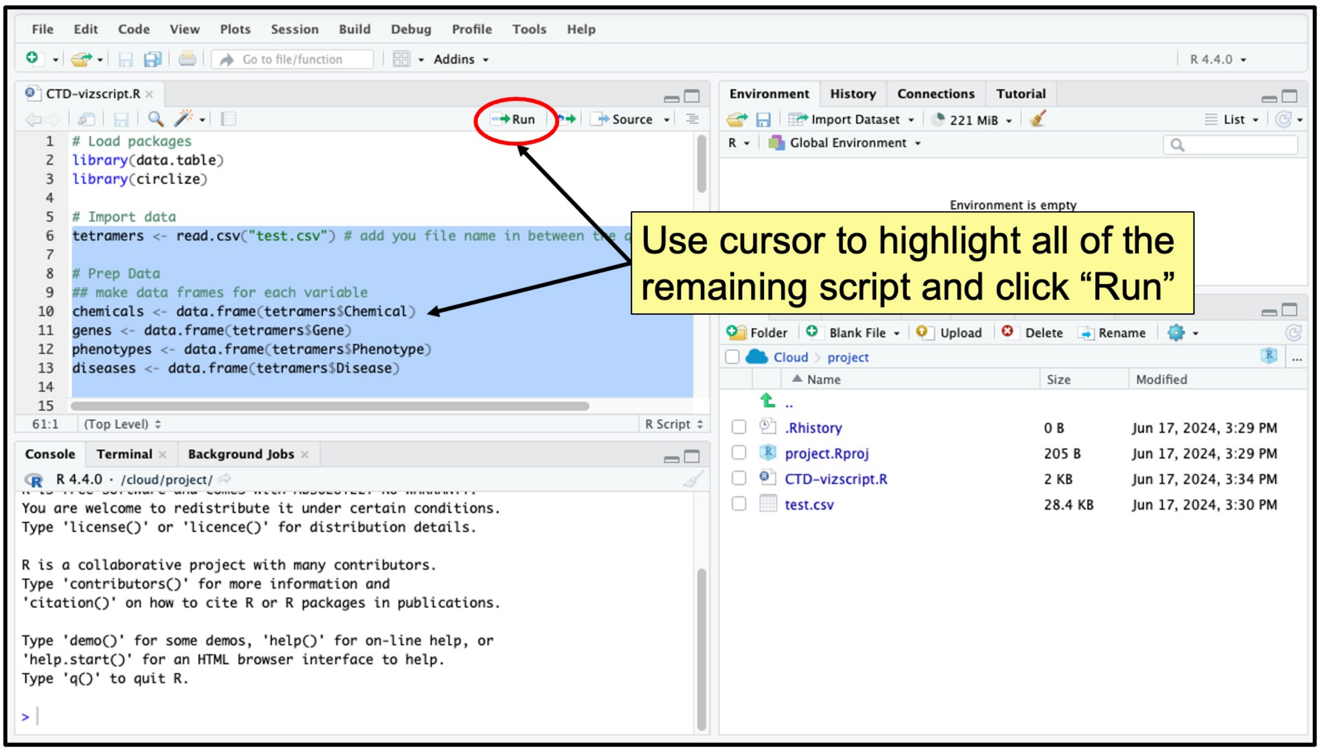
Run
# Load packages
library(data.table)
library(circlize)
# Import data
tetramers <- read.csv("test.csv") # add you file name in between
# Prep Data
make data frames for each variable
chemicals <- data.frame(tetramers$Chemical)
genes <- data.frame(tetramers$Gene)
phenotypes <- data.frame(tetramers$Phenotype)
diseases <- data.frame(tetramers$Disease)
Use cursor to highlight all of the remaining script and click "Run"

G.

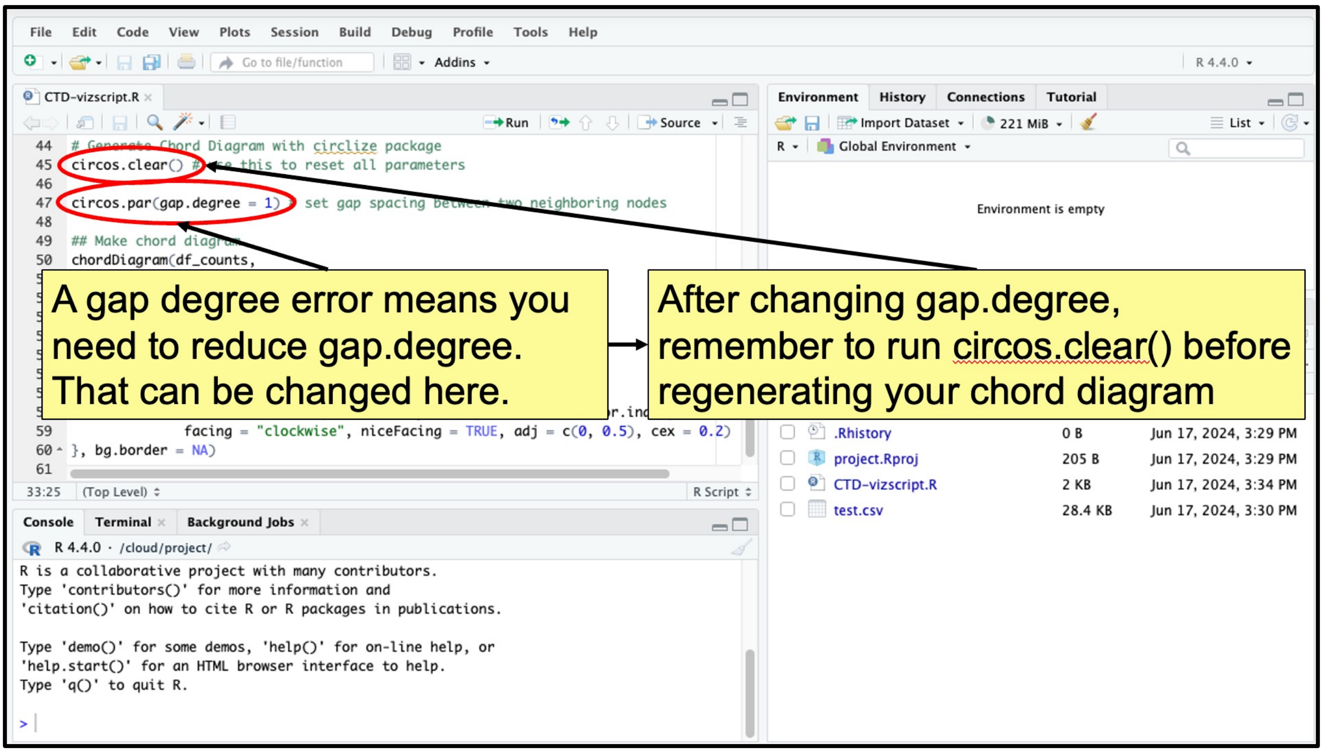
A gap degree error means you need to reduce gap.degree. That can be changed here.
After changing gap.degree, remember to run circos.clear() before regenerating your chord diagram

H.

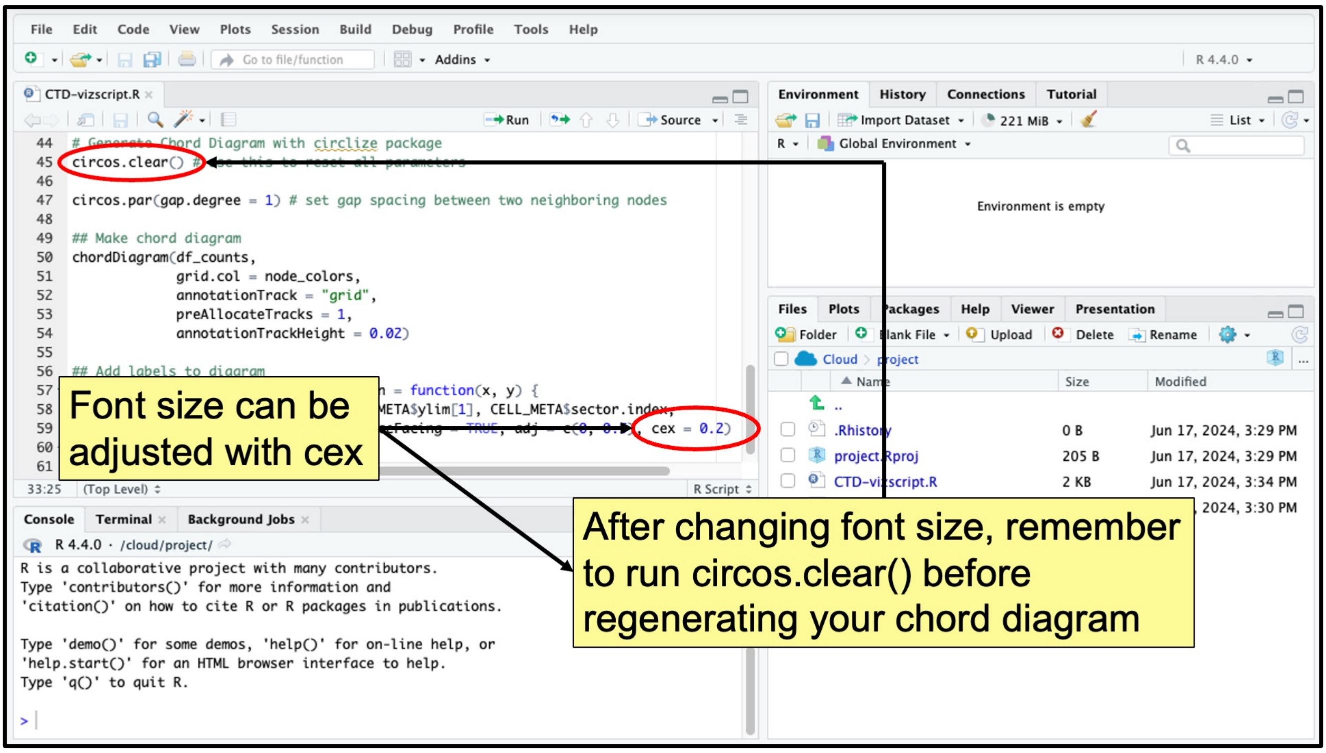
Font size can be adjusted with cex
After changing font size, remember to run circos.clear() before regenerating your chord diagram

I.

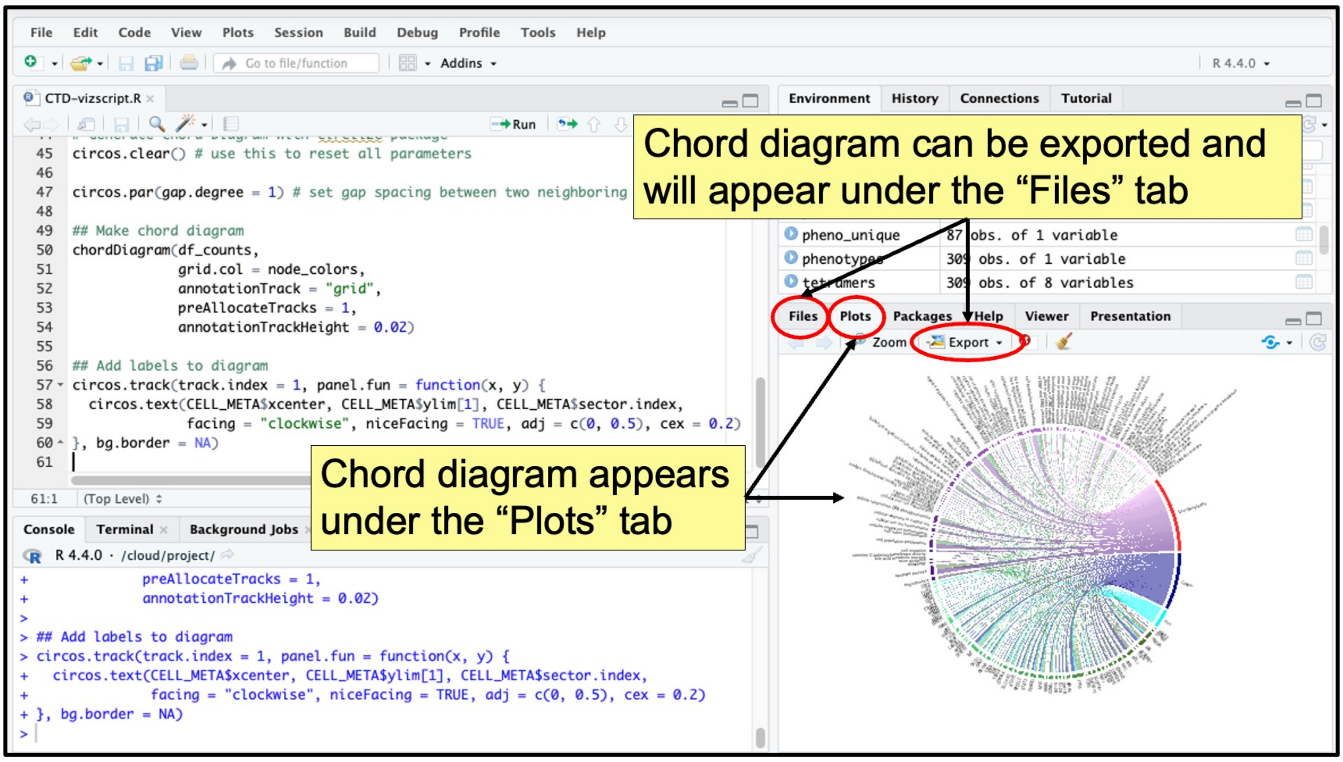
Chord diagram can be exported and will appear under the “Files” tab
Chord diagram appears under the “Plots” tab

J.

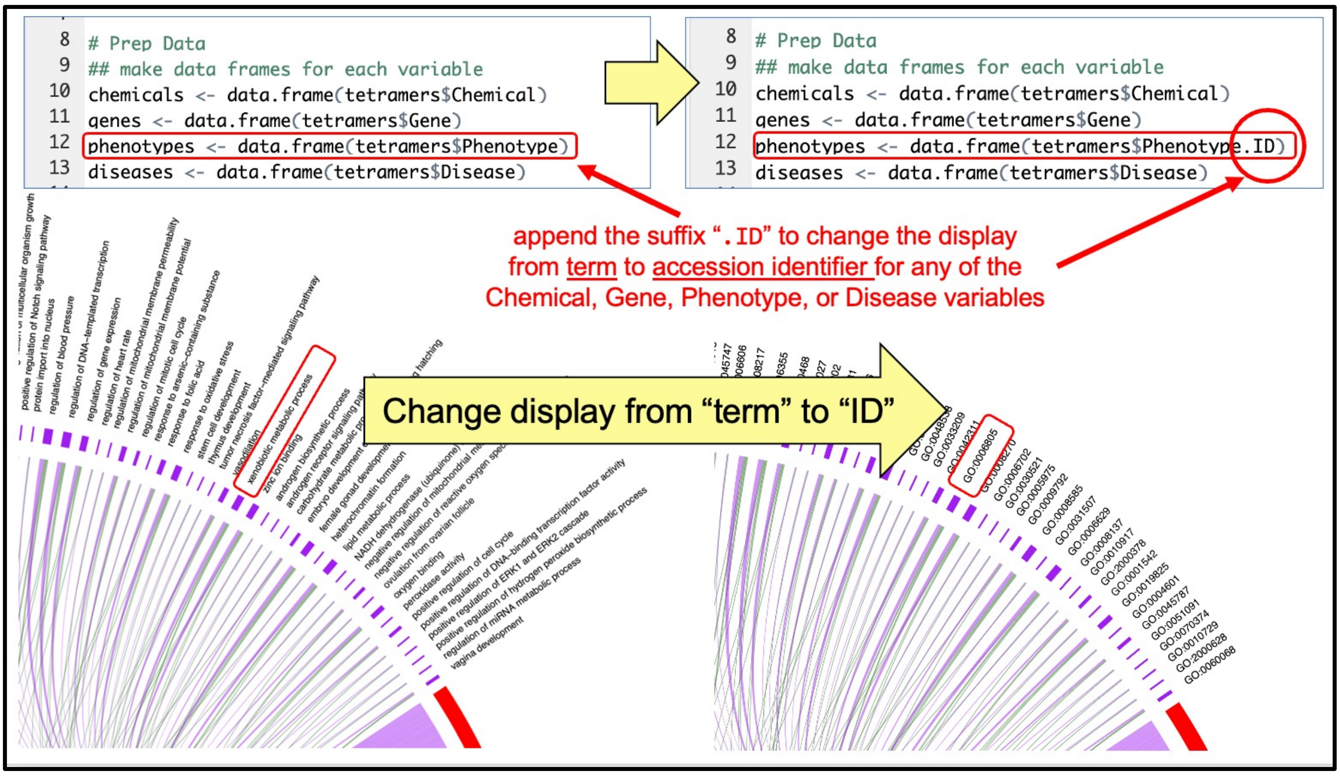

Prep Data
make data frames for each variable
chemicals <- data.frame(tetramers$Chemical)
genes <- data.frame(tetramers$Gene)
phenotypes <- data.frame(tetramers$Phenotype)
diseases <- data.frame(tetramers$Disease)
# Prep Data
make data frames for each variable
chemicals <- data.frame(tetramers$Chemical)
genes <- data.frame(tetramers$Gene)
phenotypes <- data.frame(tetramers$Phenotype.ID)
diseases <- data.frame(tetramers$Disease)
append the suffix ".ID" to change the display
from term to accession identifier for any of the
Chemical, Gene, Phenotype, or Disease variables
Change display from "term" to "ID"

K.

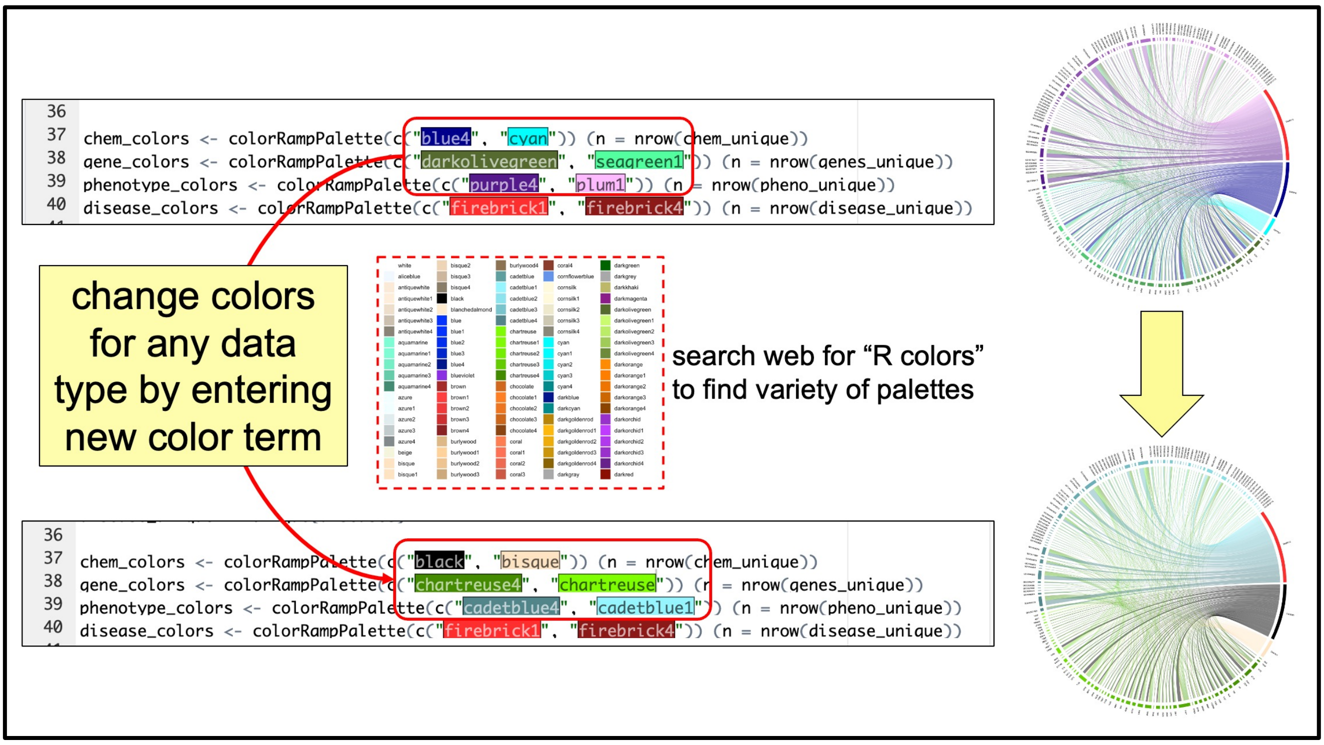

chem_colors <- colorRampPalette(c("blue4", "cyan")) (n = nrow(chem_unique))
gene_colors <- colorRampPalette(c("darkolivegreen", "seagreen1")) (n = nrow(genes_unique))
phenotype_colors <- colorRampPalette(c("purple4", "plum1")) (n = nrow(pheno_unique))
disease_colors <- colorRampPalette(c("firebrick1", "firebrick4")) (n = nrow(disease_unique))
change colors for any data type by entering new color term
search web for "R colors" to find variety of palettes
chem_colors <- colorRampPalette(c("black", "bisque")) (n = nrow(chem_unique))
gene_colors <- colorRampPalette(c("chartreuse4", "chartreuse")) (n = nrow(genes_unique))
phenotype_colors <- colorRampPalette(c("cadetblue4", "cadetblue1")) (n = nrow(pheno_unique))
disease_colors <- colorRampPalette(c("firebrick1", "firebrick4")) (n = nrow(disease_unique))

L.

```
# Load packages
library(data.table)
library(circlize)

# Import data
tetramers <- read.csv("/path/to/file.csv")# add you file name in between the quotation marks

# Prep Data
## make data frames for each variable
chemicals <- data.frame(tetramers$Chemical)
genes <- data.frame(tetramers$Gene)
phenotypes <- data.frame(tetramers$Phenotype)
diseases <- data.frame(tetramers$Disease)

## Combine into dimer relationships
chem_gene <- cbind(chemicals, genes)
colnames(chem_gene) <- c("start", "end")

gene_pheno <- cbind(genes, phenotypes)
colnames(gene_pheno) <- c("start", "end")

pheno_disease <- cbind(phenotypes, diseases)
colnames(pheno_disease) <- c("start", "end")

## Combine into one data frame
cgpd <- rbind.data.frame(chem_gene, gene_pheno, pheno_disease)

## Add count values
df_counts <- setDT(cgpd)[,list(Count=.N),names(cgpd)]

## Lists for setting colors
chem_unique <- unique(chemicals)
genes_unique <- unique(genes)
pheno_unique <- unique(phenotypes)
disease_unique <- unique(diseases)

chem_colors <- colorRampPalette(c("blue4", "cyan")) (n = nrow(chem_unique))
gene_colors <- colorRampPalette(c("darkolivegreen", "seagreen1")) (n = nrow(genes_unique))
phenotype_colors <- colorRampPalette(c("purple4", "plum1")) (n = nrow(pheno_unique))
disease_colors <- colorRampPalette(c("firebrick1", "firebrick4")) (n = nrow(disease_unique))

node_colors <- c(chem_colors, gene_colors, phenotype_colors, disease_colors)

# Generate Chord Diagram with circlize package
circos.clear() # use this to reset all parameters

circos.par(gap.degree = 1) # set gap spacing between two neighboring nodes

## Make chord diagram
chordDiagram(df_counts,
             grid.col = node_colors,
             annotationTrack = "grid",
             preAllocateTracks = 1,
             annotationTrackHeight = 0.02)

## Add labels to diagram
circos.track(track.index = 1, panel.fun = function(x, y) {
  circos.text(CELL_META$xcenter, CELL_META$ylim[1], CELL_META$sector.index,
             facing = "clockwise", niceFacing = TRUE, adj = c(0, 0.5), cex = 0.2)
}, bg.border = NA)
```

enter name of tetramer file

add ".ID" suffix to variable to change display from term to identifier

change color palettes

change gap size

"cex": change font size
